# Influence of Knowledge Management Practices on Entrepreneurial and Organizational Performance: A Mediated-Moderation Model

**DOI:** 10.3389/fpsyg.2020.577106

**Published:** 2020-12-03

**Authors:** Cai Li, Sheikh Farhan Ashraf, Fakhar Shahzad, Iram Bashir, Majid Murad, Nausheen Syed, Madiha Riaz

**Affiliations:** ^1^School of Management, Jiangsu University, Zhenjiang, China; ^2^Lyallpur Business School, Government College University Faisalabad, Faisalabad, Pakistan; ^3^Government College Women University, Faisalabad, Pakistan; ^4^Ghazi University, Dera Ghazi Khan, Pakistan

**Keywords:** knowledge management practices, dynamic capability, opportunity recognition, organizational performance, entrepreneurial performance, mediated-moderated model

## Abstract

This study aims to identify the influence of knowledge management practices on the entrepreneurial and organizational performance with the mediating effect of dynamic capabilities and moderating role of opportunity recognition. Data were gathered from 486 entrepreneurs and applied a structural equation model to test the hypotheses. We found that knowledge management practices have a positive and significant influence on dynamic capabilities, as well as have a significant impact on entrepreneurial and organizational performance. Moreover, results indicated that dynamic capabilities partially mediate in the relationship between knowledge management practices on entrepreneurial and organizational performance. Furthermore, the relationship between knowledge management practices with entrepreneurial and organizational performance strengthening by opportunity recognition. Further, implications and limitations were discussed in the paper.

## Introduction

With the rapid development in the knowledge-based economy, knowledge is considered an important measure to create prosperity and success (Abubakar et al., 2019). Knowledge is the best driving force for entrepreneurial and organizational performance and its success (Zaim et al., 2019). According to Wahda (2017) knowledge is the essential element of an organization for achieving a competitive advantage and maximum outcome. Knowledge management is defined as the explicit and effective management of important knowledge and its related practices of identification and its exploitation (Ngah et al., 2016). Effective knowledge resources make up knowledge capability among organizations with the help of knowledge sharing, knowledge creation, innovativeness, and knowledge absorption. Therefore, when these resources merged it determine the knowledge management practices which ultimately turn into the relationship with organizational performance (Alaarj et al., 2016).

Meanwhile, Butt et al. (2019) argue that organizations effort to look for means that support the workforce of knowledge resources to accomplish with the organization’s challenges in a competitive market as well as enhanced the entrepreneurial and organizational performance. Prior researchers indicate that knowledge management practices have progressively become an interest of topic in all areas of business studies and provide a significant role in the entrepreneurial and organizational success because of its growing awareness in the society (Tang, 2017). Therefore, Antunes and Pinheiro (2020) suggested that knowledge management practices would help in the development of small and medium enterprises (SME’s) and their activities so they become more strong and effective to stay longer. Looking into previous literature researchers examined the role of knowledge management practices on organizational performance and found that knowledge management positively related to organizational and business performance (Cerchione and Esposito, 2016; Serrat, 2017; Abuaddous et al., 2018).

Moreover, knowledge-based theory (KBT) explains that when knowledge management practices are effectively and efficiently managed, it develops unique capabilities that contribute to enhanced organizational performance by innovation (Kane, 2017). Therefore, organizations with superior knowledge management practices are likely to achieve organizational performance (Lopes et al., 2017; Shujahat et al., 2019). Akhavan et al. (2016) state that knowledge management practices such as knowledge sharing, knowledge acquisition, and knowledge application contributes to innovation which helps to improve organizational performance.

Furthermore, Byukusenge and Munene (2017) explain that knowledge sharing is an activity through knowledge skills, information is exchanged among people, peers, friends, or with in the organizations. Moreover, Centobelli et al. (2019) specified that innovative capacity refers to the innovation that involves the transformation of an effect into a reality that develops a new product and service that meets the needs and demands of the customers in the organizations. Researchers Santoro et al. (2018) explained that capacity as the organization’s ability to value, integrate, and apply new knowledge for improving the organizational performance. However, the relationship between knowledge sharing, innovative capacity, and absorptive capacity and organizational performance has been examined in the prior literature in the context of Western culture (Lopes et al., 2017).

Furthermore, existing studies suggested that dynamic capability playing a vital role in achieving organizational and business firm performance through sensing, knowledge sharing, and reconfiguring (Mardani et al., 2018; Antunes and Pinheiro, 2020). Prior researchers confirmed that dynamic capability had a direct and indirect positive influence on firm performance (Lin and Wu, 2014). Numerous researchers found that dynamic capability had a positive effect on organizational performance (Hung et al., 2010). Each of these studies examined the dynamic capability as a predictor variable to measure business and organizational performance and the relationship between knowledge management practices and its impact on organizational and entrepreneurial performance is under-explored. Therefore, it is necessary to identify the direct effect of knowledge management practices and the indirect effect of dynamic capability on entrepreneurial and organizational performance.

The gap of the study consists of four perspectives. Firstly, this study covers the existing gap in the literature of knowledge management practices such as knowledge sharing, innovative capacity, and absorptive capacity on organizational and entrepreneurial performance, because no empirical study is so far available on this relationship. Secondly, this study measures the performance of SME entrepreneurs using dynamic capability as a mediator because the significance of the SME sector is increasing gradually. Thirdly, most of the previous studies focused on the other sectors as well as examined the role of knowledge management practices on business performance (Hung et al., 2010; Protogerou et al., 2012; Gholami et al., 2013) and taken innovation as a mediator variable in the relationship between organizational performance and other factors such as organizational learning, entrepreneurial orientation (Hartono and Halim, 2014; Ferreira et al., 2020a). Therefore, the relationship between knowledge management practices using dynamic capability as a mediator on entrepreneurial and organizational performance of SMEs is the motivation of this study. Fourthly, the direct relationship of dynamic capability on organizational and entrepreneurial performance is defined in the literature (Ambrosini and Bowman, 2009). It is seen in the previous researches the relationship between opportunity recognition and dynamic capability on entrepreneurial and organizational performance is neglected by the researchers because opportunity recognition realizes an idea, capability that matches well with a particular target market to improve business performance. Thus, this study takes opportunity recognition as a moderating variable in the relationship between dynamic capabilities, entrepreneurial and organizational performance.

## Literature Review and Hypotheses Development

### Knowledge Management

Researchers believe that firms can stand out in one or more value-added disciplines; it can achieve unique competitive advantages and excellent organizational performance (Torabi and El-Den, 2017). Knowledge management is likely to be a value-added method, more actively using knowledge and expertise to create value and improve organizational efficiency (Rašula et al., 2012). Organizations with a higher level of knowledge management capabilities are more likely to increase the competitiveness of an entrepreneur by collecting, organizing, and transforming knowledge to implement (Shujahat et al., 2019). Therefore, knowledge management practices play an important role not only in the firm’s performance but also lead to entrepreneurial performance. The process of knowledge management operation in an organization is complex and the entrepreneurs are managing, respectively. Thus, this study focuses on the key practices which the organizations acquire and use to improve their knowledge.

### Relationship Between Knowledge Sharing Capacity, Dynamic Capability, Entrepreneurial and Organizational Performance

In the current era of a knowledge-based economy, knowledge plays an important role in driving the value of an organization. Individuals with valued knowledge help to achieve and extend the organizational performance that ultimately contributes to the sustainability of the organizations (Ha and Lo, 2018). Therefore, organizations with a lack of knowledge sharing capacities not performed well in competitive markets. Prior researches stated that entrepreneurs participated in the development and sharing of valuable knowledge, that can not only improve entrepreneurial performance as well as enhance the organizational performance (Ohemeng and Kamga, 2020). Knowledge sharing capacity assists in problem-solving, adopting new technology, creating an invention, and enhancing the dynamic capabilities of an organization (Ali et al., 2019).

The knowledge-sharing capacity of an entrepreneur develops the dynamic capability for getting competitive advantages (Liao et al., 2007). The researchers argued that knowledge sharing helps the dynamic capability of an individual and organization to develop new products, engage the entrepreneur to absorb the change, show willingness for competitive advantages (Carmeli et al., 2013; Kang and Lee, 2017). Moreover, Lin and Wu (2014) explored that dynamic capability is the combination of designed structure and learning of different activities, which helps the entrepreneur and organization in daily routine work. Dynamic capability helps in managing the inner capacities of an organization and assists in performance. Therefore, knowledge management is not enough to enhance performance until considering knowledge sharing as a dynamic capability in relation to entrepreneurial and organizational performance (Rafique et al., 2018). Therefore, this study posited that;

**H1a:** Knowledge sharing capacity has a positive influence on dynamic capability.

**H1b:** Knowledge sharing capacity has a positive influence on entrepreneurial performance.

**H1c:** Knowledge sharing capacity has a positive influence on organizational performance.

### Relationship Between Innovative Capacity, Dynamic Capability, Entrepreneurial and Organizational Performance

Innovative capacity is considered as an important factor to innovate something new or different (Furman et al., 2002). In the context of innovative capacity, the use of skills to create new ideas with an association of vision and capabilities (Lawson and Lorenz, 1999). Every organization plans to start a new corporation with a unique approach, the challenge is not only to discover an excellent idea but also to invent an opportunity that helps the entrepreneur to build with innovative capacity (Halkos and Skouloudis, 2018). There are less empirical researches proves that innovative capacity and organizational performance growth parallel (Hernández-Perlines et al., 2019). Gieske et al. (2016) argue that innovative capacity is based on human and capital resources; it also depends on the overall infrastructure of the organization and the combination of a proactive and innovative environment. The process of commercialization of an organization has interacted through innovative capacity, which directly affects and increases the percentage of organizational performance in the market.

The absorption of external knowledge prepares the entrepreneur to increase the innovative capacity (Wu et al., 2017). Innovative capacity determined the organizational culture, leadership characteristics, procedure of product invention, and the use of strategies in launching new products with organizational performance (Proksch et al., 2017). Many studies have been conducted to consider the role of innovative capacity and its relation with dynamic capability in organizational performance (Najmi et al., 2018). Organizations with innovative capacity and proactive behavior change the business environment to improve performance (Zhou et al., 2019). Furthermore, researchers explored that innovative capacity raises the energy level of an organization, which positively influences on organizational performance (Fainshmidt et al., 2016).

Ferreira et al. (2020b) described innovation is the process to improve and launch a new product in the market, enhance product quality and productivity through the development of the manufacturing process and its adoption. García-Sánchez et al. (2018) explained that as the level of innovative capacity becomes higher; it gives an edge to the entrepreneurial performance by using dynamic capabilities. The entrepreneur utilizes dynamic capabilities to absorb innovation for competitive advantages. Moreover, the innovative capacity differentiates entrepreneurs and organizations across the market due to their competitive dynamic capabilities. The innovative capacity and dynamic capability associate to attain the performance in a professional setting (Liu et al., 2018). Considering the innovative capacity as a vital dynamic capability lead toward the entrepreneurial and organizational performance of textile-based SMEs, this study hypothesized that;

**H2a:** Innovative capacity has a positive impact on dynamic capability.

**H2b:** Innovative capacity has a positive influence on entrepreneurial performance.

**H2c:** Innovative capacity has a positive influence on organizational performance.

### Relationship Between Absorptive Capacity, Dynamic Capability, Entrepreneurial and Organizational Performance

Absorptive capacity assists the entrepreneurs in understanding and utilizing valuable information, to build marketing strategies, which generate long term financial profit and increase the performance (Kale et al., 2019). The significant relationship between absorptive capacity and dynamic capability has been proved by Latukha and Veselova (2019) and further included the process of evaluation and adaptation for entrepreneurial performance in an organization. Liu et al. (2020) proposed that imminent absorptive capacity and comprehended absorptive capacity are essential, rather than adequate, and to attain competitive organizational benefits, both expected and comprehended capability plays a significant role in enhancing the performance. Absorptive capability is a blend of potential absorptive capability and comprehended absorptive capability, and is known as potential competency, which permits an organization to increase, assimilate, integrate, transfer and utilize new knowledge for the organizational and entrepreneurial performance (Chaudhary and Batra, 2018).

Furthermore, Ahn et al. (2016) proposed that the firm’s absorptive capacity plays a beneficial role in the research and development activities and organizational learning of the firms. Therefore, the firms with a high level of absorptive capacity lead the firms to enhance their innovation performance. Additionally, Xue et al. (2019) asserted that the firm’s absorptive capacity is considered to be critical to the firm’s innovative capabilities. Ince et al. (2016) endorsed the positive influence of absorptive capacity on dynamic capability, which improves entrepreneurial skills. The absorptive capacity allows entrepreneurs or organizations to absorb internal and external knowledge, which is necessary to gain ideas and implications for performance strategies. Few studies focused on the firms’ absorptive capacity in deriving technological information from external means and how it contributes to organizational skills and activities (Verma et al., 2017; Chaudhary, 2019). Absorptive capacity is not only a base for organizational performance, but other factors are also involved, such as entrepreneurial performance (Rangus and Slavec, 2017). Therefore, absorptive capacity has been considered as an important part of dynamic capability, which boosts the performance of textile-based SMEs.

**H3a:** Absorptive capacity has a positive impact on dynamic capability.

**H3b:** Absorptive capacity has a positive influence on entrepreneurial performance.

**H3c:** Absorptive capacity has a positive influence on organizational performance.

### Relationship Between Dynamic Capability, Entrepreneurial and Organizational Performance

Dynamic capability is the part of the entrepreneurial restructuring and environmental changes, which is directly linked with its performance. In high-tech firms, the dynamic capabilities of an entrepreneur are the most reliable and sound source for taking advantage (Jiang et al., 2018). Raza et al. (2018) dynamic capabilities cover sensing, reconfiguring, and seizing capability of a performance organization. The dynamic capabilities of an organization guide in utilizing valuable resources during the performance (Zhou et al., 2019). Moreover, dynamic capability help to innovate a new product accepts to create and show its willingness to achieve competitive advantage through knowledge sharing behavior. In some organizations, employees are afraid to share knowledge with entrepreneurs and other colleagues to hinder the progress of other co-workers (Falasca et al., 2017). Prior researchers believed that, once the discouraging knowledge sharing behavior establish in an organization environment, it will be unfavorable, difficult to change (Ha and Lo, 2018; Ferreira et al., 2020a).

Looking into previous studies resource-based theory explored the relationship between the dynamic capability of an entrepreneur and entrepreneurial performance (Battisti and Deakins, 2017; Wang and Kim, 2017). The dynamic capability of an entrepreneur assists in facing new challenges, exploring opportunities to maintain and develop organizational performance. The decision-making power and dynamic capability of an organization with market strategies enhance innovative capacity, which assists in-process and technological innovation (Rafique et al., 2018). The researcher suggested that procedure of attaining, developing, distributing, and providing services from dealers to customers with dynamic organizational capabilities enhance organizational performance (Pezeshkan et al., 2016). Moreover, the organization requires peripheral resources to supplement the inefficiency of their internal skills and actions with dynamic capability for organizational performance (Bamel and Bamel, 2018).

Now a day’s many organizations are working on people as a resource for performance. The employee-driven force, with dynamic capability in an organization, plays a significant impact on competitive advantages and organizational performance (Braganza et al., 2017). Organizations with dynamic capability overcome the competitor threats and block the competitor’s actions (Likoum et al., 2020); it minimizes the expected competitor’s actions with potential adverse in organizational performance and facilitates the entrepreneurs and organization with idea creation. Therefore, the following hypotheses are proposed:

**H4a:** Dynamic capability has a positive impact on entrepreneurial performance.

**H5a:** Dynamic capability has a positive impact on organizational performance.

### Mediating Effect of Dynamic Capability

Prior researchers argued that dynamic capability has a positive impact on organizational performance (Xing et al., 2020). Dynamic capability helps to develop a new product by knowledge sharing capacity of the entrepreneur within the organization (Wang and Kim, 2017). Knowledge sharing increases the knowledge resource with a considerable role of the dynamic capability to achieve a competitive advantage (Kang and Lee, 2017). Researchers explored that innovative capacity raises the energy level of an organization, which positively influences performance (Proksch et al., 2017). Moreover, organizations with a higher level of innovative capacity are more prone to perform well, and in a better position to recognize market opportunities (Torabi and El-Den, 2017). The absorptive capacity of an entrepreneur absorbs the innovative technology and makes it feasible for an organization to accumulate the resources for objectives and competitive advantages (Kale et al., 2019). Furthermore, in a similar context, absorptive capacity, and dynamic capability are found fundamental to organizational success (Ferreira et al., 2020b). Organizations with a higher absorptive capacity assist in learning from competitors with firm dynamic capabilities as well as demonstrate the knowledge in organizations for better performance (Latukha and Veselova, 2019).

There is a considerable role in dynamic capability as a mediator between organizational performance and knowledge management practices. The proper utilization of dynamic capability is acquired knowledge, innovative, and absorptive capacities lead the performance of an entrepreneur and organization (Likoum et al., 2020). Therefore, this study incorporates the mediating role of dynamic capability in the relationship between knowledge management practices such as knowledge sharing, innovative, and absorptive capacity with entrepreneurial and organizational performance. Therefore, the following hypotheses are proposed:

**H4b:** Dynamic capability mediates the relationship between knowledge sharing capacity and entrepreneurial performance.

**H5b:** Dynamic capability mediates the relationship between knowledge sharing capacity and organizational performance.

**H4c:** Dynamic capability mediates the relationship between innovative capacity and entrepreneurial performance.

**H5c:** Dynamic capability mediates the relationship between innovative capacity and organizational performance.

**H4d:** Dynamic capability mediates the relationship between absorptive capacity and entrepreneurial performance.

**H5d:** Dynamic capability mediates the relationship between absorptive capacity and organizational performance.

### Relationship Between Entrepreneurial and Organizational Performance

Entrepreneurial performance is concerned with risk-taking and decision-making attitude, product invention for the organization, and market innovation (Kantur, 2016). Entrepreneurial performance associated with the new values and creativity, time, resources, risks, and another ingredient toward organizational performance (Miao et al., 2017). The prior studies show that entrepreneurial performance can lead the firm performance (Chavez et al., 2017; Al-Henzab et al., 2018). Moreover, prior studies argued that entrepreneurial performance is an essential factor for the long term survival and development of the organization (Hartono and Halim, 2014). Al-Dhaafri et al. (2016) found that entrepreneurial performance always has a positive influence on organizational performance and can help organizations to achieve competitive advantages. Furthermore, Filser and Eggers (2014) examined the role of entrepreneurial performance on organizational performance researching different countries such as Austria, Liechtenstein, and Switzerland, which found that entrepreneurial performance significantly influenced SME’s development. Thus, entrepreneurial performance enabling the achievement of organizational performance and propose the following hypothesis.

**H6:** Entrepreneurial performance has a positive impact on organizational performance.

### The Moderating Role of Opportunity Recognition in the Relationship Between Entrepreneurial and Organizational Performance

Opportunity recognition proposed that the cognitive of different entrepreneur’s results are different in the entrepreneurial process and performance (Hmieleski and Baron, 2008). Hasan et al. (2016) discussed the mediating role of opportunity recognition in association with entrepreneurial performance and found it as a critical factor in enhancing entrepreneurial performance. Furthermore, a large number of scholars suggested that self-made strategies of an entrepreneur play a significant role in the process of opportunity recognition (Bagheri, 2017; Ploum et al., 2018). However, due to less focus by researchers on this crucial factor, we incorporate opportunity recognition in this study to measure its impact on the relationship between dynamic capability and entrepreneurial performance. Therefore, competitive advantages are important for entrepreneurs and also impact organizational performance until unless dynamic capabilities put through, and capabilities are important for performance (Teece et al., 2016).

Opportunity recognition is to recognize the capabilities to attain the best source from the market for competitive advantages and entrepreneurial performance (Teece, 2016). Entrepreneurial opportunities are renowned through circumstances that new goods, services, raw materials, and procedures could be offered and commercialized at advanced value than the production budget. There is a deficiency in opportunity recognition, concerning entrepreneurial performance (George et al., 2016), and the efficacious entrepreneur always chooses appropriate opportunity with competences (Kim et al., 2018), formerly and subsequently business ventures leads to the successful entrepreneurial performance. Opportunity recognition plays a vital role in entrepreneurial performance.

The opportunity for organizational performance, positive entrepreneur behavior, dynamic capabilities, market knowledge, positioning of services provide more opportunities to acquire the market to grow and survive (Jantunen et al., 2005). The researchers argue that organizations with dynamic capabilities obtain more competitive advantages than other firms, and opportunity recognition gives a chance for better performance in product development and organizational performance (Chirico and Nordqvist, 2010; Swoboda and Olejnik, 2016). However, there is less focus on SME’s empirical research related to the moderating role of opportunity recognition and its drivers in smaller organizations. Ferreira et al. (2020a) focused on the dynamic organizational capabilities in small organizations with opportunities for competitive advantages.

Sanz-Velasco (2006) argued that market interaction and entrepreneurs’ life experiences related to the market, industrial knowledge, and resources should be considered for opportunity recognition. The researchers proposed that an opportunity may have an impression of vaguely distinct market needs, which means that potential consumers may or may not have the capability to articulate their demands and interests (Roundy et al., 2018; Li et al., 2020b). The identification of the needs of a customer might lead to a prompt appearance of opportunity recognition, which is a result of better organizational performances (Hu et al., 2018).

Besides, the researchers suggested that market potential influences the opportunity recognition in the process of product development (Obschonka and Hahn, 2018; Neneh, 2019). Therefore, the idea of entrepreneurship is related to the process of evaluation, discovery, exploration, sources, and recognition of opportunities that highly influence the entrepreneurial and organizational performance (Campos, 2017). Thus, this study postulates that better opportunity recognition would lead to higher organizational and entrepreneurial performance and formulate the following hypothesis:

**H7a:** Opportunity recognition positively moderates the relationship between dynamic capability and entrepreneur performance.

**H7b:** Opportunity recognition positively moderates the relationship between dynamic capability and organizational performance.

## Materials and Methods

### Sample and Data Collection

**FIGURE 1:**
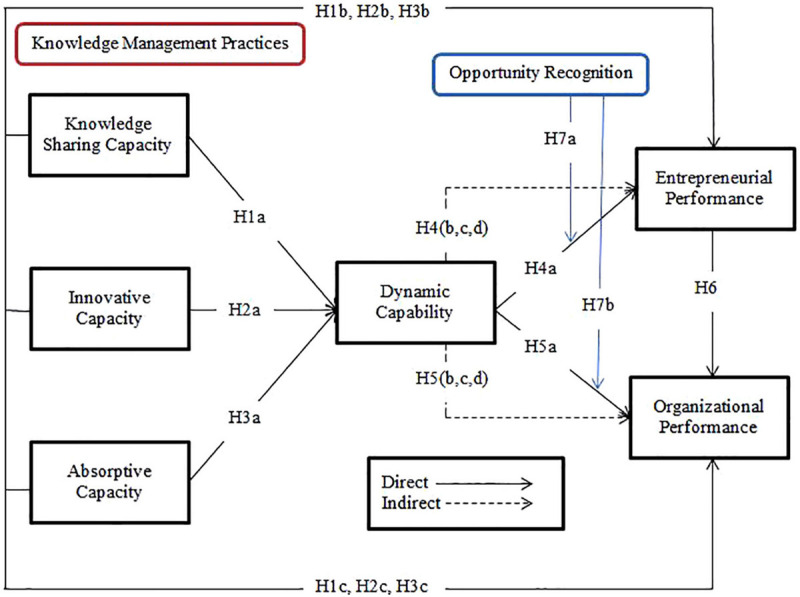
Conceptual model.

The nature of this study was cross-sectional and data were collected through a convenience sampling technique. Figure 1 shows the conceptual model of the study. The target population was the SME’s of Pakistan because SME’s were considered as the backbone industry of Pakistan. Moreover, we selected big cities such as Lahore, Faisalabad, Sheikhupura, Karachi, Multan, and Sialkot of Pakistan for data collection. To avoid the issue of common method bias (Podsakoff et al., 2012), we collected data in two rounds using the time-lag approach. In the first round, we collected data for knowledge management practices and dynamic capability measures. In the second round, we collected data for entrepreneurial and organizational performance and opportunity recognition. However, due to the unavailability of registered SME’s in Pakistan, we contacted small and medium chambers of commerce of every city to provide the list of SMEs, after getting the list from the chamber we contacted the SME’s owners through emails and personal visits.

Furthermore, we distributed 600 paper-pencil questionnaires to the respondents who positively respond to us on email and personal visits. We ensured them that this research is purely for academic purposes and the information will be confidential. The original draft of the questionnaire was in English and Urdu language because some of the SME’s owners were illiterate. Finally, in the initial screening, we received 508 questionnaires with a participation rate was 84.6% and 22 responses were dropped due to missing data. Thus, the final sample size was 486 responses. Among the valid responses, all the respondents were male and the age of respondents was starting from 18 years to 47 years and above. The highest age range of respondents was 33–39 (32.30%). Additionally, the highest work experience of the respondent was 1–5 years (26.13%) and the region of SMEs was Faisalabad, Lahore, Sialkot, Sheikhupura, Karachi, and Multan. The highest response rate was from Faisalabad 119 (24.48%) and the lowest response rate was from Sialkot 31 (6.37%).

### Measures

To ensure the realistic and effective content of the research model, a structured questionnaire was compiled, and all exogenous variables were constructed and operationalized from the existing literature of knowledge sharing capacity, innovative capacity, absorptive capacity, dynamic capability, and opportunity recognition, entrepreneurial and organizational performance. To measure the 41 constructs, we used a 5-point Likert scale ranging from 1 strongly disagree to 5 strongly agree to quantify the results.

### Knowledge Sharing Capacity

To measure knowledge sharing capacity five items were adapted from the study of Hsu et al. (2007). This scale is widely accepted and used by previous researchers (Davenport and Prusak, 1998; Keikha, 2018). A sample item, “I frequently participate in knowledge sharing activities.”

### Innovative Capacity

To assess innovative capacity we have adopted five measurement constructs from the study of Hurley and Hult (1998). A sample item “risk-taking is encouraged in our firm.”

### Absorptive Capacity

To measure absorptive capacity four items were used developed by Leal-Rodríguez et al. (2014). A sample item “our firm regularly considers the consequences of changing market demand in terms of new ways to provide services.”

### Dynamic Capability

A dynamic capability was measured using two dimensions exploration and exploitation, with 3 items each. This scale was adapted from the study of Atuahene-Gima (2005). This scale was used by previous researchers (Ferreira et al., 2020a). A sample item for exploration “acquired manufacturing technologies and skills entirely new the firm.” A sample item off exploitation “upgraded current knowledge and skills for familiar products and technologies.”

### Opportunity Recognition

The five measurement items for opportunity recognition taken from the study of Kuckertz et al. (2017). A sample item “my organization always alert to business opportunities.”

### Entrepreneurial Performance

To measure entrepreneurial performance, we used eleven items scale developed by Colbert et al. (2008). A sample item “entrepreneurs: forms goals, allocates resources to meet them, and monitors progress toward them.”

### Organizational Performance

To examine organizational performance, four items were adopted from the study (García-Morales et al., 2008). A sample item “return on assets.”

## Results

### Data Analysis Technique

We used the partial least square structural equation modeling (PLS-SEM) technique to test the measurement model and structural model results. The Smart-PLS3 software is used to cover the flaws in the data and bring fluency in data results. This software is also used to estimate the causal and empirical model relationship between the variables as well as examine the correlation between constructs, respectively (Hair et al., 2010). Nowadays this software is considered as a silver bullet in the field of management science research and used by several researchers to test the hypotheses results (Hair et al., 2011; Li et al., 2020a).

### Measurement of Model

The fitness of the model was assessed through reliability and validity analysis. Table 1 shows the values for Cronbach’s alpha (CA), rho_A, the average value extracted (AVE), and composite reliability (CR). The values of convergent validity should be higher than the thrush hold values; rho_A ≥ 0.7, CR ≥ 0.8, AVE ≥ 0.50, and CA ≥ 0.80. Therefore, it is seen that all the constructs were above a threshold value and acceptable range as benchmark suggested by Nunally and Bernstein (1978). Moreover, the values for Cronbach’s alpha was 0.936–0.953, values for AVE was 0.666–0.839, value for rho_A was 0.934–0.954, and values of CR was 0.952–0.964.

**TABLE 1:** Construct reliability and validity.

**Constructs**	**Cronbach’s alpha**	**rho_A**	**Composite reliability**	**Average variance extracted (AVE)**
AC	0.936	0.940	0.954	0.839
DC	0.945	0.950	0.957	0.787
EP	0.950	0.951	0.956	0.666
IC	0.936	0.937	0.952	0.798
KSC	0.953	0.954	0.964	0.842
OP	0.933	0.934	0.952	0.833
OR	0.942	0.943	0.954	0.775

*KSC, Knowledge Sharing Capacity; IC, Innovative Capacity; AC, Absorptive Capacity; DC, Dynamic Capability; OR, Opportunity Recognition; and EP, Entrepreneurial Performance.*

### Discriminant Validity

Discriminant validity was measured using two criteria’s Fornell–Larcker and Heterotrait-Mono-Trait Ratio (HTMT). Table 2 shows the results of Fornell–Larcker criteria, as per this criterion the square root of AVE is called discriminant validity (Fornell and Larcker, 1981). Therefore, it is observed in Table 2 the values were higher than the correlations was discriminant validity. Furthermore, HTMT criteria were also applied to analyze the discriminant validity. As per this criterion, the values for HTMT should be less than one (Henseler et al., 2015). It is seen in Table 3 all the values of HTMT are up to the threshold value. Thus, there was no issue in discriminant validity.

**TABLE 2:** Fornell-larcker criterion.

	**AC**	**DC**	**EP**	**IC**	**KSC**	**OP**	**OR**
AC	0.916						
DC	0.403	0.887					
EP	0.411	0.394	0.816				
IC	0.317	0.350	0.421	0.893			
KSC	0.523	0.407	0.432	0.427	0.918		
OP	0.406	0.419	0.455	0.390	0.479	0.913	
OR	0.221	0.345	0.358	0.174	0.233	0.378	0.880

*Value with diagonals is the square root of the AVE. Value under diagonals is correlations. KSC, Knowledge Sharing Capacity; IC, Innovative Capacity; AC, Absorptive Capacity; DC, Dynamic Capability; OR, Opportunity Recognition; and EP, Entrepreneurial Performance.*

**TABLE 3:** HTMT ratio criterion.

	**AC**	**DC**	**EP**	**IC**	**KSC**	**OP**	**OR**
AC							
DC	0.428						
EP	0.432	0.410					
IC	0.337	0.370	0.446				
KSC	0.551	0.427	0.453	0.450			
OP	0.432	0.445	0.481	0.416	0.507		
OR	0.233	0.366	0.378	0.186	0.245	0.402	

*KSC, Knowledge Sharing Capacity; IC, Innovative Capacity; AC, Absorptive Capacity; DC, Dynamic Capability; OR, Opportunity Recognition; and EP, Entrepreneurial Performance.*

### Structural Model

The structural model was measured through a bootstrapping test and the level of significance. The fitness of the structural model was assessed through standardized root means square residual (SRMR). According to Henseler et al. (2015) a value of a good model should have a <0.08 of SRMR value. Thus, the value for SRMR was 0.043 which below the threshold value. Moreover, the structural model explained *R*^2^ 26.5% variance in dynamic capability, 25.2% variance in entrepreneurial performance, and 30.6% variance in organizational performance. According to Chin (1998) desired values of *R*^2^ must be greater than 0.1 or zero. Hence, the structural model results of *R*^2^ were greater than 0.1 values which show the positive predictive significance of the model.

### Testing of Hypotheses

**FIGURE 2:**
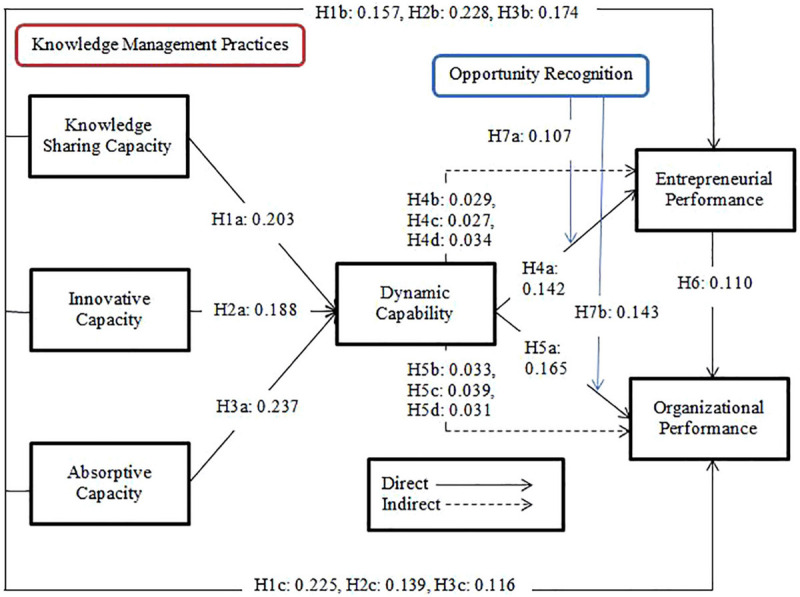
Structural model.

The results of the hypotheses were shown in Table 4 and Figure 2. This study proposed H1a KSC positively influence on DC and the results indicate that KSC has a positive and significant impact on dynamic capability (β = 0.203^∗∗^, *t* = 4.567, and *p* < 0.001). Moreover, we predicted H1b KSC positively influence on EP and the findings illustrate that KSC positively related to the EP (β = 0.157^∗∗^, *t* = 3.116, and *p* < 0.002). Meanwhile, we proposed H1c KSC positively effect on OP and the outcome indicates that KSC has a positive impact on OP (β = 0.225^∗∗^, *t* = 4.149, and *p* < 0.001). Thus, H1a, H1b, and H1c were accepted.

**TABLE 4:** Path coefficients (direct effects).

**Hypotheses**	**Original sample (O)**	**Sample mean (M)**	**S.D**	**T statistics (|O/STDEV|)**	***P* values**
H1a: KSC → DC	0.203**	0.202	0.044	4.567	0.001
H1b: KSC → EP	0.157**	0.157	0.051	3.116	0.002
H1c: KSC → OP	0.225**	0.226	0.054	4.149	0.00
H2a: IC → DC	0.188**	0.189	0.042	4.470	0.001
H2b: IC → EP	0.228**	0.227	0.044	5.192	0.001
H2c: IC → OP	0.139**	0.136	0.044	3.191	0.001
H3a: AC → DC	0.237**	0.235	0.049	4.829	0.001
H3b: AC → EP	0.174**	0.172	0.048	3.641	0.001
H3c: AC → OP	0.116**	0.116	0.045	2.588	0.010
H4a: DC → EP	0.142**	0.143	0.047	3.020	0.003
H5a: DC → OP	0.165**	0.167	0.047	3.540	0.001
H6: EP → OP	0.110**	0.110	0.053	2.063	0.039

*p* < 0.001**, *p* < 0.05*. KSC, Knowledge Sharing Capacity; IC, Innovative Capacity; AC, Absorptive Capacity; DC, Dynamic Capability; OR, Opportunity Recognition; and EP, Entrepreneurial Performance.

Furthermore, we predicted H2a IC positively influence on DC and results explain that IC has a positive and significant influence on DC (β = 0.188^∗∗^, *t* = 4.470, and *p* < 0.001). Moreover, we proposed that H2b IC positively affects EP and the findings indicate that IC has a positive and significant impact on EP (β = 0.228^∗∗^, *t* = 5.192, and *p* < 0.001). Besides, we predicted H2c IC positively influence on OP and the results illustrate that IC has a positive effect on OP (β = 0.139^∗∗^, *t* = 3.191, and *p* < 0.001). Hence, H2a, H2b, and H2c were supported.

Additionally, we assumed that H3a AC positively influences on DC and the findings indicate that AC has a positive and significant impact on DC (β = 0.237^∗∗^, *t* = 4.829, and *p* < 0.001). Moreover, we proposed H3b AC positively effects EP and the results show that AC has a positive and significant influence on EP (β = 0.174^∗∗^, *t* = 3.641, and *p* < 0.001). Furthermore, we predicted H3c AC positively impact on OP and findings illustrate that AC also has a positive and significant impact on OP (β = 0.116^∗∗^, *t* = 2.588, and *p* < 0.010). Therefore, H3a, H3b, and H3c were accepted.

Lastly we, predicted H4a that DC positively effects on EP and results indicate that DC positively influence on EP (β = 0.142^∗∗^, *t* = 3.020, and *p* < 0.003). Moreover, we proposed H5a DC positively effect on OP and findings show that DC has a positive and significant impact on OP (β = 0.165^∗∗^, *t* = 3.540, and *p* < 0.001). Furthermore, we predicted H6 EP positively leads to OP and the outcomes explain that EP has a positive and significant influence on OP (β = 0.110^∗∗^, *t* = 2.063, and *p* < 0.039). Thus, H4a, H5a, and H6 were also supported.

### Mediating Effect of Dynamic Capability

We tested the mediating effect of dynamic capability in the relationship between knowledge sharing capacity, innovative and absorptive capacity with entrepreneurial and organizational performance and results were shown in Table 5. We proposed H4b DC mediates positively between KSC and EP and we found that DC has a positive indirect effect in the relationship between KSC and EP (β = 0.029^∗∗^, *t* = 2.385, and *p* < 0.017). Moreover, we predicted H4c DC positively mediates between IC and EP and we found that DC has a positive indirect influence in the relationship between IC and EP (β = 0.027^∗∗^, *t* = 2.398, and *p* < 0.017). Furthermore, we supposed H4d DC mediates the AC and EP and the results indicate that DC has a positive and significant indirect impact in the relationship between AC and EP (β = 0.034^∗∗^, *t* = 0.013, and *p* < 0.011).

**TABLE 5:** Mediation analysis (indirect effects).

**Hypotheses**	**Original sample (O)**	**Sample mean (M)**	**S.D**	**T statistics (|O/STDEV|)**	***P* Values**
H4b: KSC → DC → EP	0.029**	0.029	0.012	2.385	0.017
H4c: IC → DC → EP	0.027**	0.027	0.011	2.398	0.017
H4d: AC → DC → EP	0.034**	0.034	0.013	2.546	0.011
H5b: KSC → DC → OP	0.033**	0.034	0.012	2.737	0.006
H5c: AC → DC → OP	0.039**	0.039	0.013	2.902	0.004
H5d: IC → DC → OP	0.031**	0.032	0.012	2.507	0.012

*p* < 0.001**, *p* < 0.05*. KSC, Knowledge Sharing Capacity; IC, Innovative Capacity; AC, Absorptive Capacity; DC, Dynamic Capability; OR, Opportunity Recognition; and EP, Entrepreneurial Performance.

Additionally, we predicted H5b DC positively mediates the relationship between KSC and OP and we found that DC has a positive indirect influence in the relationship KSC and OP (β = 0.033^∗∗^, *t* = 2.737, and *p* < 0.006). Besides, we proposed H5c DC mediates positively between AC and OP and findings show that DC has a positive indirect effect in the relationship between AC and OP (β = 0.039^∗∗^, *t* = 2.902, and *p* < 0.004). Meanwhile, we proposed H5d DC positively mediates between IC and OP and we found that DC also has an indirect effect in the relationship between IC and OP (β = 0.031^∗∗^, *t* = 2.507, and *p* < 0.012). Hence, H5b, H5c, H5d were accepted.

### The Moderating Role of Opportunity Recognition

**FIGURE 3:**
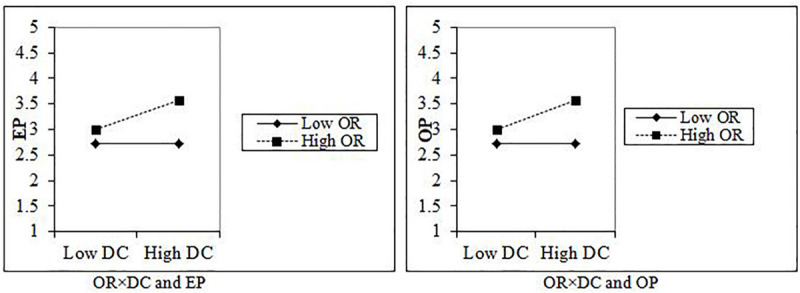
Interaction of OP and DC with EP and OR.

The moderating role of OR was also testified with the help of structural model results. Table 6 and Figure 3 show the moderating impact of OR in the relationship between DC with EP and OP. Moreover, we tested H7a OR to have a significant and positive moderation effect in the relationship between DC and EP. The results indicate that OR strengthening the relationship between DC and EP (β = 0.107^∗∗^, *t* = 4.135, and *p* < 0.001). Furthermore, we predicted H7b OR in the relationship between DC and OP and the findings show that OR strengthening the positive and significant role in the relationship between DC and OP (β = 0.143^∗∗^, *t* = 3.221, and *p* < 0.001). Therefore, H7a and H7b were accepted.

**TABLE 6:** Moderating effects.

**Hypotheses**	**Original sample (O)**	**Sample mean (M)**	**S.D**	**T statistics (|O/STDEV|)**	***P* Values**
H7a: OR*DC → EP	0.107**	0.109	0.026	4.135	0.000
H7b: OR*DC → OP	0.143**	0.138	0.045	3.221	0.001

*p* < 0.001**, *p* < 0.05*. DC, Dynamic Capability; OR, Opportunity Recognition; and EP, Entrepreneurial Performance.

### Common Method Bias and Multicollinearity Test

Common method bias and variance inflation factor (VIF) factors (multicollinearity) were also performed. We used Harman’s test to find out the common method bias in the data. According to Harman (1976) if all the factors merged in principle rotated matrix and the initial eigenvalue explaining >50% of the variance. There is an issue of common method bias. Therefore, we performed the analysis using principle rotated matrix and the factors emerged from factor analysis and the first factor of initial eigenvalue explaining 40.24% of the total variance. Thus, there is no issue of common method bias in the data. Furthermore, the VIF test also performed. As suggested by Aiken et al. (1991) value of VIF should be between the 5 to 10 were acceptable and if the values were above 10 there is an issue in multicollinearity. The output of Table 7 shows that there is no issue of multicollinearity in the data.

**TABLE 7:** Cross loadings.

	**AC**	**DC**	**EP**	**IC**	**KSC**	**OP**	**OR**
AC1	**0.911**	0.388	0.435	0.293	0.515	0.405	0.223
AC2	**0.912**	0.340	0.351	0.254	0.447	0.373	0.197
AC3	**0.922**	0.362	0.334	0.293	0.472	0.350	0.199
AC4	**0.919**	0.382	0.377	0.318	0.477	0.354	0.188

DC1	0.317	**0.853**	0.367	0.297	0.352	0.343	0.292
DC2	0.344	**0.785**	0.254	0.253	0.272	0.334	0.296
DC3	0.372	**0.881**	0.384	0.306	0.376	0.397	0.301
DC4	0.366	**0.937**	0.384	0.346	0.387	0.424	0.342
DC5	0.375	**0.918**	0.343	0.311	0.383	0.347	0.300
DC6	0.369	**0.937**	0.352	0.339	0.384	0.378	0.307

EP1	0.323	0.251	**0.764**	0.328	0.326	0.305	0.283
EP10	0.344	0.302	**0.800**	0.344	0.363	0.359	0.245
EP11	0.315	0.319	**0.818**	0.318	0.342	0.393	0.248
EP2	0.349	0.252	**0.813**	0.359	0.332	0.347	0.286
EP3	0.345	0.303	**0.839**	0.370	0.364	0.391	0.337
EP4	0.309	0.328	**0.810**	0.359	0.373	0.360	0.327
EP5	0.358	0.360	**0.777**	0.300	0.348	0.384	0.280
EP6	0.396	0.490	**0.846**	0.369	0.413	0.428	0.273
EP7	0.343	0.272	**0.870**	0.375	0.343	0.372	0.314
EP8	0.308	0.323	**0.846**	0.329	0.342	0.376	0.293
EP9	0.284	0.299	**0.785**	0.324	0.315	0.351	0.325

IC1	0.310	0.326	0.409	**0.842**	0.403	0.338	0.149
IC2	0.313	0.310	0.369	**0.870**	0.381	0.342	0.140
IC3	0.274	0.312	0.368	**0.924**	0.389	0.369	0.134
IC4	0.262	0.318	0.375	**0.912**	0.395	0.369	0.172
IC5	0.252	0.291	0.356	**0.915**	0.335	0.320	0.184

KSC1	0.513	0.390	0.405	0.431	**0.947**	0.460	0.202
KSC2	0.467	0.379	0.406	0.437	**0.927**	0.451	0.209
KSC3	0.490	0.381	0.381	0.386	**0.919**	0.430	0.215
KSC4	0.410	0.343	0.375	0.336	**0.873**	0.415	0.204
KSC5	0.516	0.374	0.415	0.367	**0.919**	0.439	0.238

OP1	0.399	0.402	0.443	0.366	0.475	**0.905**	0.324
OP2	0.342	0.389	0.434	0.338	0.426	**0.891**	0.290
OP3	0.359	0.370	0.392	0.362	0.420	**0.923**	0.378
OP4	0.379	0.366	0.390	0.357	0.423	**0.930**	0.387
OR1	0.169	0.260	0.298	0.160	0.195	0.324	**0.903**
OR2	0.214	0.349	0.320	0.139	0.215	0.352	**0.902**
OR3	0.248	0.291	0.329	0.174	0.229	0.286	**0.844**
OR4	0.241	0.302	0.344	0.152	0.240	0.357	**0.846**
OR5	0.178	0.302	0.297	0.156	0.177	0.346	**0.914**

*KSC, Knowledge Sharing Capacity; IC, Innovative Capacity; AC, Absorptive Capacity; DC, Dynamic Capability; OR, Opportunity Recognition; and EP, Entrepreneurial Performance. Bold values with diagonals are factor loadings.*

## Discussion

This study investigates the impact of dynamic capability as a mediator and the role of opportunity recognition as a moderator between dynamic capability with entrepreneurial and organizational performance. The study path coefficient provides empirical support to the proposed hypotheses and found significant findings with *p*-value < 0.05 and *t*-value > 2. The results support our hypothesis H1a knowledge sharing capacity predicts greater DC, which supported the explanation and consistent with the prior studies of Chirico and Nordqvist (2010) and Ferreira et al. (2020b). The dynamic capability is helpful incompetency to figure, integrate, and reconfigure internal and external capability to enhance rapid change in the environment. The result of H1b offers that knowledge sharing capacity has a positive relationship with entrepreneurial performance and the findings are in line with the previous researchers commented on by Hsu et al. (2007) and Liao et al. (2007). The result of H1c confirms that knowledge sharing capacity has a significant impact on organizational performance and commented with the studies of Torabi and El-Den (2017) and Ali et al. (2019).

The result of H2a proposed that innovative capacity influenced dynamic capability and the outcome is consistent with the prior studies of Hung et al. (2010) and Ferreira et al. (2020b). The result of H2b offers that innovative capacity has a positive impact on entrepreneurial performance and the finding is similar to a prior study of Jantunen et al. (2005). The outcome of H2c proposed that innovative capacity positively influenced organizational performance and finding is matched with the previous study of Furman et al. (2002).

Moreover, the finding of H3a found that absorptive capacity positively affects dynamic capability and the results are consistent with existing studies (Chaudhary and Batra, 2018; Kale et al., 2019). Meanwhile, the result of H3b suggested that absorptive capacity positively influenced entrepreneurial performance, and finding is matched with the study of Kang and Lee (2017). The result of H3c supported that absorptive capacity has a positive impact on organizational performance and the finding is in line with the previous researcher (Chaudhary, 2019).

The finding of H4a dynamic capability has a positive influence on entrepreneurial performance. This result is consistent with the prior scholar (Ferreira et al., 2020b). Furthermore, the H5a result stated that dynamic capability positively and significantly related to the organizational performance, and finding is matched to the existing study of Fainshmidt et al. (2016). Besides, the result of H6 suggested that entrepreneurial performance significantly influenced organizational performance, and the result of H4b stated that dynamic capability as a mediating effect in the relationship between knowledge sharing capacity and entrepreneurial performance. This finding is similar to previous researchers (Hsu et al., 2007; Swoboda and Olejnik, 2016; Torabi and El-Den, 2017). The result of H4c confirms that innovative capacity trigger dynamic capability on entrepreneurial performance and the result is consistent with (Hung et al., 2010). The result of H4d stated that dynamic capability positively mediates the relationship with absorptive capacity and entrepreneurial performance and finding is confirmed to (Ahn et al., 2016).

Additionally, the result of H5b suggested that dynamic capability positively mediates in the relationship between knowledge sharing capacity and organizational performance, and the findings are consistent with prior studies of Protogerou et al. (2012) and Teece (2016). The finding of H5c recommended that dynamic capability positively mediates in the relationship between absorptive capacity and organizational performance. This result is similar to Zhou et al. (2019). The result of H5d found that dynamic capability positively mediates the relationship between innovative capacity and organizational performance. This finding is matched to (Bamel and Bamel, 2018).

Lastly, the result of H7a found that opportunity recognition positively moderates the relationship between dynamic capability and entrepreneurial performance. The finding stated that opportunity recognition strengthens the positive and significant moderation effect on the relationship between dynamic capability and entrepreneurial performance. This output is consistent with prior studies of Sanz-Velasco (2006) and Roundy et al. (2018). Moreover, the result of H7b suggested that opportunity recognition moderates in the relationship between dynamic capability and organizational performance. This result is also in line with the prior findings of researchers (Jiang et al., 2018; Ploum et al., 2018).

## Conclusion

This research extends the existing literature by exploring the importance of knowledge management practices, dynamic capabilities, and opportunity recognition to increase SME’s entrepreneurial and organizational performance. Numerous researches have been devoted to evaluating the SME’s performance and recognized the role of knowledge management practices with dynamic capabilities to achieve appropriate results. Therefore, the dynamic capabilities of SMEs in the term or knowledge management practices via capabilities and opportunities play a vital role in entrepreneurial and organizational performance. The finding of this research indicated that knowledge management practices regulate the SME’s entrepreneurial and organizational performance with the significant values of beta coefficient, *t*-values, and *p*-values. Furthermore, results suggested that dynamic capabilities play a vital role in SME’s performance, and opportunity recognition moderates the relationship between dynamic capability with entrepreneurial and organizational performance. These arguments narrate how knowledge management practices assist entrepreneurs and organizations in performance, which may positively affect on unemployment and economic growth in a country.

### Practical Implications

This study has some practical implications for industry practitioners, the SME sector, and researchers in the field of entrepreneurship and organizational performance. Firstly, the study contributes to the scientific literature of SME’s performances, knowledge management capacities, dynamic capabilities, and opportunities. For a better understanding of government and non-government textile-based SME sectors, recommended deriving from this research result, which is beneficial in reducing the graph of failure business. Secondly, this study suggested that textile-based SMEs with less performance will get much assistance through this research. Thirdly, this study helps SMEs to establish a more effective way to transfer knowledge in an organization to develop a strong environment for achieving organizational goals-against competitors. It is important for the organizational operation and emerging economies because the organization faces a shortage of internal and external information, which affects the SME’s performance. Fourthly, with the help of dynamic capabilities, SMEs develop the organizational and entrepreneurial quality across the organizational boundaries. Furthermore, this study also brings riven literature on knowledge management capacities into a broader perspective for SME’s performances.

### Limitations and Future Research Directions

The study has few limitations, which need to be acknowledged. The data was collected from one source or the same source. The limitation for the cross-sectional nature of data also exists, and for future research, for researchers, longitudinal data is recommended. For future research direction, this model will assist in multi-disciplinary SMEs, to raise the level of entrepreneurial and organizational performance in Pakistan. The precise and better conclusion for researchers may consider demographics, government policies, and regulation for SMEs as control variables. Here, another limitation related to the study, the sample population was bound to the gender and capture 100% of males due to the selected region. The business was based on male category businesses. This research finding may be affected due to gender discrimination. So, for future research replication to the current study should consider the gender composition. Finally, the proposed model of research was tested on Pakistani male entrepreneurs and organizations running through the male businessman. However, for future recommendation, the research may consider more and different industries, including big-size sample data with male and female entrepreneurs. This research may replicate and increase in the research model for applicability to find.

Furthermore, future researchers also conduct a similar pattern of research in a different time frame. As it is aforementioned that knowledge and innovation capacity is not constant it grew and may enhance as the context evolved with development. Hence, the knowledge and learning ability of a person may vary as time passes. It’s the main course of reason to suggest future researchers conduct a longitudinal study for the spectrum presented in this research.

## Data Availability Statement

The raw data supporting the conclusions of this article will be made available by the authors, without undue reservation.

## Ethics Statement

Ethical review and approval was not required for the study on human participants in accordance with the local legislation and institutional requirements. Written informed consent for participation was not required for this study in accordance with the national legislation and the institutional requirements.

## Author Contributions

SA conceived the idea and developed a proposed model to discuss. All authors provided critical feedback and helped to shape the research, analysis, and manuscript. CL led the whole project and direct in all steps, she refined the idea and directed all authors to move on. SA and IB collectively worked to design the research plan. They encouraged FS and MR to investigate and pilot testing. Further all authors participated in data collection. MM and NS developed the theory and SA helped him to perform the computations. FS and IB verified the analytical methods. SA and MM took the lead in writing the manuscript and supervised the findings of this work. All authors discussed the results and contributed to the final manuscript. All authors provided critical feedback and helped to shape the research, analysis, and manuscript. At the final stage and revision of the manuscript MM, NS, and MR prepared the document according to the mutually decided pattern which has considered as the best presentation of prescribed research design.

## Conflict of Interest

The authors declare that the research was conducted in the absence of any commercial or financial relationships that could be construed as a potential conflict of interest.
